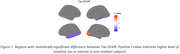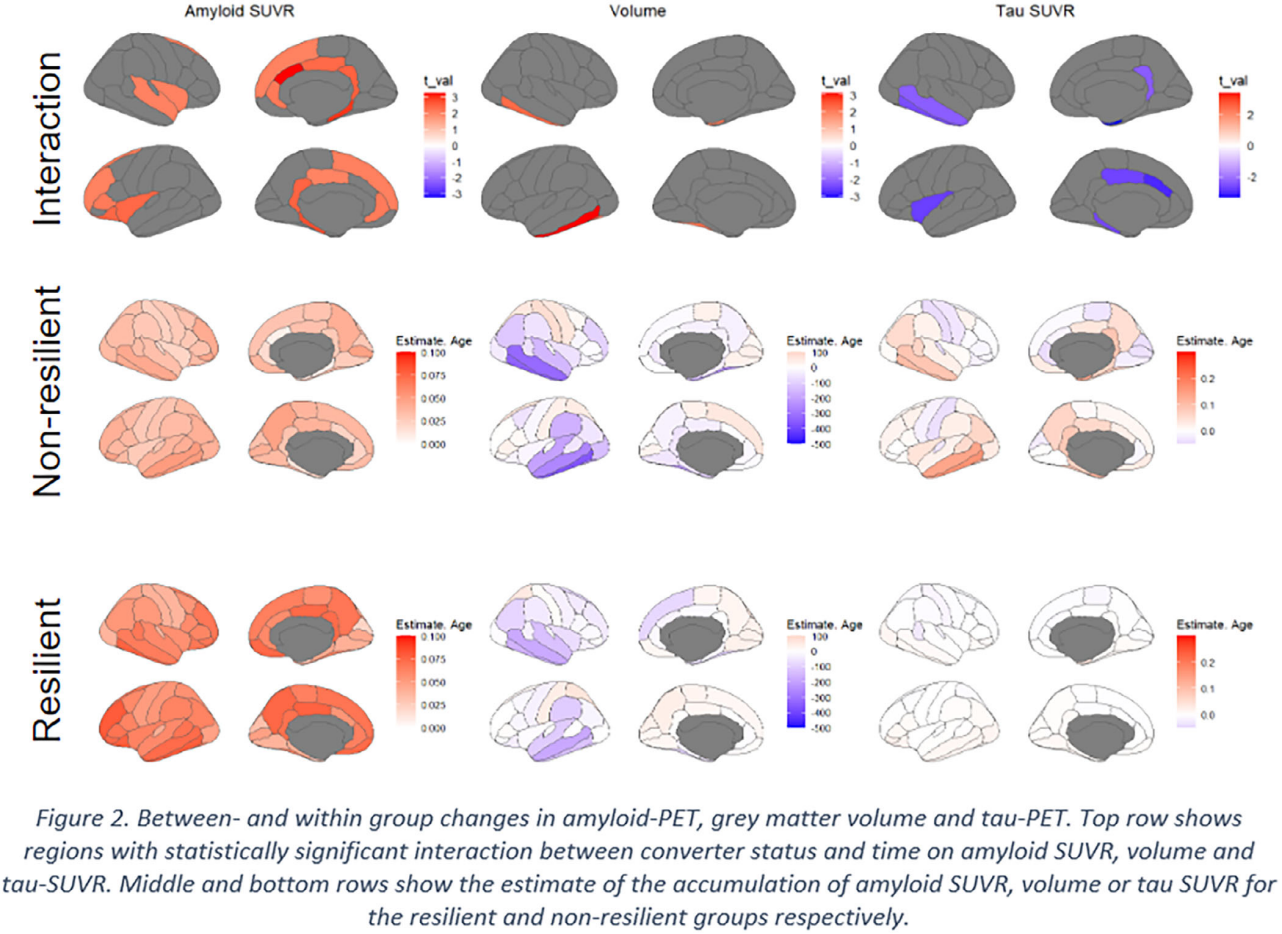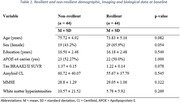# Pathways towards Cognitive resilience in the context of amyloid pathology: a longitudinal matched study design

**DOI:** 10.1002/alz.090764

**Published:** 2025-01-09

**Authors:** Pablo Aguillar, Muge Akinci, Eleni Palpatzis, Alexandre Bejanin, Eider M Arenaza‐Urquijo

**Affiliations:** ^1^ ISGlobal, Barcelona, ‐ Spain; ^2^ Universitat Pompeu Fabra, Barcelona, Barcelona Spain; ^3^ Sant Pau Memory Unit, Hospital de la Santa Creu i Sant Pau, Biomedical Research Institute Sant Pau, Barcelona, Barcelona Spain; ^4^ Universitat Pompeu Fabra, Barcelona Spain; ^5^ ISGlobal, Barcelona, Cataluña Spain; ^6^ Sant Pau Memory Unit, Hospital de la Santa Creu i Sant Pau, Biomedical Research Institute Sant Pau, Universitat Autònoma de Barcelona, Barcelona Spain; ^7^ ISGlobal ‐ Barcelona Institute for Global Health, Barcelona, Catalunya/Barcelona Spain

## Abstract

**Background:**

Cognitive resilience can be defined as better‐than‐expected cognitive performance in the context of Alzheimer’s disease (AD) pathologies or increased AD risk. We investigated pathways associated with cognitive resilience trajectories in amyloid positive (A+) and/or APOE4 cognitively unimpaired (CU) older adults including brain resilience, resistance to AD pathologies and vascular pathology.

**Method:**

We included 534 CU ADNI participants with available cognitive data, longitudinal amyloid‐PET ( [^18^F]florbetaben and [^18^F]florbetapir) and structural MRI (gray matter volumes) and, as ubset with tau‐PET ( [^18^F]AV1451) (n = 287) and white matter hyperintensities (n = 467) volume data (n = 534). We performed a one‐to‐one matched design, aligning participants by their baseline amyloid levels, MMSE scores, and age within an average follow‐up time of 6 years (68.18 ±32 months). We identified 44 CU participants ‐ defined as cognitive resilient ‐ who were A+ and/or APOE4 carriers and remained CU and 44 CU A+ matched participants who converted to MCI or AD (non‐resilient). To assess brain resilience, resistance to pathologies and vascular pathways, at baseline, t‐test were performed with gray matter volumes, amyloid (n = 44), tau (n = 12) and WM measurements (n = 27) as outcomes of interest. Longitudinally, linear mixed effect models were used to examine the interaction between cognitive resilient status (resilient vs non) and time on regional amyloid and tau deposition, and gray matter volume.

**Result:**

At baseline, cognitive resilient participants were characterized by lower tau burden in temporal regions (Figure 1). Longitudinally, cognitive resilient participants showed lower tau accumulation in medial temporal and limbic areas, including the insular and anterior cingulate cortices, and lower volume loss in fusiform, inferior temporal and entorhinal cortex (Figure 2). Unexpectedly, cognitive resilience participants showed greater amyloid increases notably in the anterior cingulate, although both groups showed increased amyloid accumulation. Sensitivity analysis adjusting by conversion time, to rule out the impact of varying accumulation rates due to disease progression, rendered similar results.

**Conclusion:**

Our results suggest that processes downstream amyloid deposition including resistance to tau and brain maintenance might be key to maintain cognitive function in the presence of amyloid pathology.